# Strategies for the Treatment of Parkinson’s Disease: Beyond Dopamine

**DOI:** 10.3389/fnagi.2020.00004

**Published:** 2020-01-31

**Authors:** Alexandre Iarkov, George E. Barreto, J. Alex Grizzell, Valentina Echeverria

**Affiliations:** ^1^Laboratorio de Neurobiología, Facultad de Ciencias de la Salud, Universidad San Sebastián, Concepción, Chile; ^2^Department of Biological Sciences, University of Limerick, Limerick, Ireland; ^3^Health Research Institute, University of Limerick, Limerick, Ireland; ^4^Department of Psychology and Neuroscience, Center for Neuroscience, University of Colorado, Boulder, CO, United States; ^5^Research & Development Service, Bay Pines VA Healthcare System, Bay Pines, FL, United States

**Keywords:** gene therapy, prevention, cotinine, stem cells, precision medicine

## Abstract

Parkinson’s disease (PD) is the second-leading cause of dementia and is characterized by a progressive loss of dopaminergic neurons in the substantia nigra alongside the presence of intraneuronal α-synuclein-positive inclusions. Therapies to date have been directed to the restoration of the dopaminergic system, and the prevention of dopaminergic neuronal cell death in the midbrain. This review discusses the physiological mechanisms involved in PD as well as new and prospective therapies for the disease. The current data suggest that prevention or early treatment of PD may be the most effective therapeutic strategy. New advances in the understanding of the underlying mechanisms of PD predict the development of more personalized and integral therapies in the years to come. Thus, the development of more reliable biomarkers at asymptomatic stages of the disease, and the use of genetic profiling of patients will surely permit a more effective treatment of PD.

## Introduction

Due to the inability to find effective preventive or curative therapies (Fox et al., 2018), epidemiological predictions for the worldwide incidence of Parkinson’s disease (PD) are not optimistic (Lees et al., 2009; AlDakheel et al., 2014; Savica et al., 2016). For example, it is projected that by the year 2040, neurodegenerative diseases will surpass cancer as the leading cause of disease-related death (Gammon, 2014). Accordingly, there is an urgent need to identify effective treatments that transcend a reduction of symptoms.

PD leads to an array of symptoms and is characterized by a progressive loss of motor functions with bradykinesia, gate alterations, and posture instability (Gelb et al., 1999; Dexter and Jenner, 2013). Other symptoms include anxiety, depression, cognitive dysfunction, hallucinations, hypophonia, micrographia, and dysphagia as well as sialorrhea, dysphagia, hyposmia, impaired color vision, bladder hyperreflexia, and abnormalities of nociception and sleep (Voon et al., 2011; Hemmerle et al., 2012; Lindqvist et al., 2012; Schapira et al., 2017b). The pathological hallmark of the disease is the presence of Lewy bodies containing increased levels of α-synuclein, neurofilaments, and ubiquitin in neuronal and glial cells across an array of brain regions (Braak et al., 1998; Kotzbauer et al., 2001; Martin et al., 2012).

While PD was first described almost 200 years ago (Parkinson, 2002), it took nearly 150 years to determine that deficiencies of the dopamine (DA) system play a leading role in the pathology’s etiology (Lees et al., 2009). In the mid-20th century, *Arvid Carlsson*, who was later awarded the Nobel Prize in Physiology and Medicine for these discoveries in 2000, found that a decrease in DA levels in the brain led to PD-like symptoms (Carlsson et al., 1957). Then, Alexander et al. (1986) described parallel afferent pathways originating from the dorsal striatum. It has since been argued that the death of DA producing neurons leads to imbalanced communication from the dopaminergic midbrain system. Inasmuch, it is believed that motor dysfunction results from altered signaling of both direct and indirect pathways to the Globus pallidus internal (GPi)/Substantia nigra pars reticulata (SNpr) regions, which significantly disrupts thalamic connectivity with the motor cortex (Joyce, 2001; Gerfen and Surmeier, 2011; Calabresi et al., 2014). Concerning the cognitive and emotional deficits, it has been shown disruption of the output from dopaminergic neurons in the ventral tegmental area (VTA; Blonder and Slevin, 2011).

Despite these discoveries, the fundamental mechanisms inducing dopaminergic cell death are still unknown (Olanow and Tatton, 1999; Han et al., 2015, 2016). Among various pools of neurons that produce DA in the central nervous system, midbrain dopaminergic neuronal lesions alone can lead to PD-like symptoms in animal models. However, there appears to be some resilience associated with DA cell death, as humans must lose 48–68% of the dopaminergic neurons of the substantia nigra pars compacta (SNc) and about 70–80% of the DA content of the striatum to experience symptomatic PD (Bernheimer et al., 1973; Fearnley and Lees, 1991).

Given that PD genesis is multifactorial and depends mainly on the age of disease onset (Marsden, 1990; Cheng et al., 2010; Okun, 2017), the prognosis is highly variable, and personalized treatment regimens are theoretically possible. While early diagnosis of PD is difficult, some display an extended period (up to 5 years) of asymptomatic development (Fearnley and Lees, 1991). This latent period of disease progression opens up broad opportunities for therapeutic intervention. Accordingly, the early detection of degenerating dopaminergic neurons opens the possibility of halting PD progression at asymptomatic stages. In this review article, we review the pathological changes associated with the functional disorganization of the frontostriatal circuit in PD and overview current efforts to develop therapeutic approaches for treating PD.

### Anatomy, Morphology and Functional Organization of the Midbrain DA System

The complexity of the dopaminergic system seems to coincide with evolutionary development given that the number, size, and distribution, as well as receptor subtypes of dopaminergic neurons in the brain, increases alongside phylogenetic complexity (Callier et al., 2003; Yamamoto and Vernier, 2011; Yamamoto et al., 2013). For example, dopaminergic terminal fields arising from midbrain clusters are more prominent and less segregated in the neocortex of primates than in rodents (Joel and Weiner, 2000; Björklund and Dunnett, 2007).

**Figure 1:**
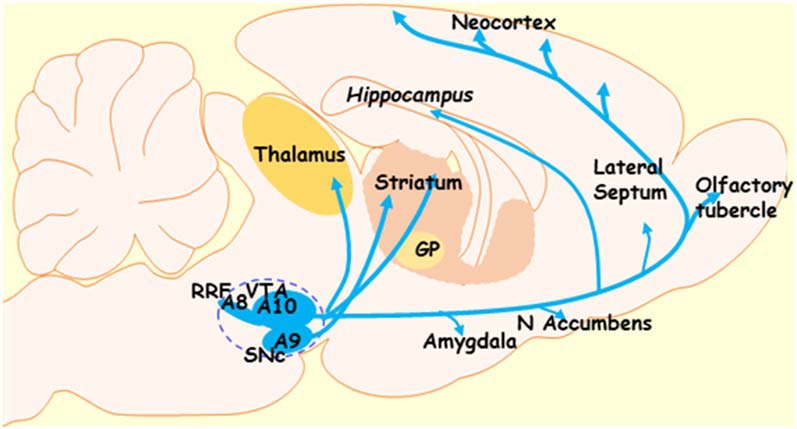
Diagram depicting the dopaminergic system of the midbrain. The dotted lines indicate the dopaminergic regions of the midbrain. GP, globus pallidus; SNc, substantia nigra pars compacta; VTA, ventral tegmental area; RRF, retrorubral field; A8, A9, and A10—clusters of dopaminergic neurons in the midbrain (RRF, SNc, and VTA clusters, respectively).

Dopaminergic neurons in the midbrain are mainly located in the SNc and VTA, although some smaller clusters have been found elsewhere, for instance, the dorsal and median raphe nuclei (Ochi and Shimizu, 1978). In a classic article by Dahlstroem and Fuxe (1964), SNc and VTA DA neurons were characterized based on their organization and projection patterns, which, in rat, can be found discrete clusters (A8, A9, and A10 see Figure 1). SNc neurons (cluster A9) innervate the dorsal and lateral striatum, thus forming a nigrostriatal pathway (Andén et al., 1964), and are necessary for the initiation and control of motor movements. Accordingly, the degeneration of this pathway is considered to be responsible for much of the motor dysfunction associated with PD. The VTA (A10) innervates the ventral striatum, nucleus accumbens, and limbic and cortical areas, and this way forms the mesolimbic and mesocortical pathways (Willner, 1991; Schott et al., 2008).

It has been documented that VTA neurons are involved in the regulation of motivation and reward as well as emotion-related behavior. Accordingly, degeneration of dopaminergic VTA neurons may underlie the development of depression and anhedonia in patients with PD (Thobois et al., 2010; Blonder and Slevin, 2011; Drui et al., 2014). Finally, the A8 cluster projects from the retrorubral field (RRF), and functionally appears related to the A10 cluster.

While the contributions from Dahlstroem and Fuxe (1964) were monumental, recent research calls this oversimplified view into question. As referred to above, organizational complexity is more significant in primates than in rats. Furthermore, the projection patterns from SNc and VTA neurons are not so refined, even within rodents. For examples, in addition to the targets listed above: (i) DA neurons of the SNc also innervate cortical and limbic regions; (ii) dopaminergic VTA neurons project to both the ventral striatum and the ventral-medial aspects of the head of the caudate-putamen; and (iii) the A8 cell group, which forms the dorsal and caudal expansion of the A9 cell group, contains cells that project into striatal, limbic, and cortical regions (Bentivoglio and Morelli, 2005; Björklund and Dunnett, 2007).

In general, midbrain DA neurons could be divided into two classes: the DA neurons located in the more dorsal tier of VTA and SNc and A8 cluster cells that are typically fusiform in appearance, with 2–5 dendrites emanating from the poles of each neuron (Björklund and Dunnett, 2007). These A8 cells are characteristically calbindin-positive and express relatively low levels of the DA transporter (DAT). In contrast, more ventrally located DA neurons are both multipolar in shape, are packed more densely, are calbindin-negative, and express higher levels of DAT. The ventral tier neurons extend (probably exclusively) to the striatum where they innervate, above all, the striosome compartment. Furthermore, many of these cells also possess prominent dendritic arborizations that spread within the zona reticulata (Björklund and Dunnett, 2007).

DA neurons of midbrain clusters have several unique morphological characteristics that may contribute to their specialized functions. For example, DA neurons appear capable of storing and releasing DA from their dendrites, permitting self-regulatory control of neurotransmitter release from nigral afferent fibers as well as to influence the activity of non-dopaminergic nigral cells (Cheramy et al., 1981). SN midbrain neurons typically have long unmyelinated axons and massive dendrites that branch into SN reticulata; the somas of these DA neurons make up much less than 1% of the total cell volume. A relatively small number of neurons provide the massive dopaminergic innervation of the striatum. It is estimated that each SN neuron can have as many as 150,000 presynaptic terminals in the striatum (Sulzer, 2007). In that regard, much is required for the normal functioning of a neuron with such a morphology. For example, each DA neuron must contain highly active axonal transport through microtubules to support metabolic and repair processes, synaptogenesis, removal of cell waste, and communication with other brain cells (Prots et al., 2013, 2018; Lu et al., 2014). In turn, each of these processes requires constant mitochondrial production of adenosine 5’-triphosphate (ATP) to assist the motor proteins dynein, kinesin, myosin, and actin (Course and Wang, 2016; Course et al., 2017; Vanhauwaert et al., 2019). Altogether, this makes the DA neurons in SN especially susceptible to mitochondrial dysfunction, and the resultant energy deficits could contribute mightily to DA-related impairments, such as those occurring in PD (Horowitz et al., 2011; Venkateshappa et al., 2012; Burbulla et al., 2017; Prots et al., 2018).

### Mitochondrial Dysfunction: A Pivotal Pathological Mechanism of Parkinson’s Disease

Mitochondria are complex cytosolic organelles of eukaryotic cells whose primary function is the generation of cellular energy in the form of ATP by oxidative phosphorylation. Mammalian mitochondria contain between 2 and 10 mitochondrial DNA (mtDNA) molecules encoding 22 transfer RNAs, two ribosomal RNAs, and 13 polypeptides, each of which is part of the respiratory chain and the oxidative phosphorylation system (Schapira, 1994). The mitochondrial respiratory chain contains four protein complexes that form the site of oxidative phosphorylation. This site is responsible for NADH and FADH2 oxidation, co-occurring with the movement of protons from the matrix into the intermembrane space. This movement produces an electrochemical gradient denoted as mitochondrial membrane potential (ΔΨm). This gradient stimulates the ATP synthase to reduce molecular oxygen and synthesize ATP. This step is fundamental in aerobic metabolism and constitutes the primary provider of ATP at the final stage of cellular respiration (Schapira, 1994; Videira and Castro-Caldas, 2018). Nevertheless, the biological function of mitochondria goes far beyond energy production and includes the metabolism of lipids and amino acids and the support of intermediate metabolic pathways, such as the Krebs cycle.

Mitochondria also play numerous regulatory actions outside of metabolism, which underscores the importance of maintaining optimal mitochondrial function. For example, mitochondrial cells regulate calcium homeostasis, remove free radicals, and control programmed neuron death (Keane et al., 2011; Franco-Iborra et al., 2016). In that regard, mitochondrial dysregulation or dysfunction can lead to an array of cellular and neuronal circuit perturbations. One common source of such disruptions is oxidative stress and, cyclically, neuroinflammation. Oxidative stress occurs as a result of high levels of unstable radicals, also called reactive oxygen and nitrogen species (RONS). These radicals can, rapidly and somewhat indiscriminately, alter proximal molecular structures through chained reduction-oxidation (redox) reactions (Betteridge, 2000). Proper mitochondrial-dependent cellular function is particularly threatened by oxidative stress due to multiple unique features of mitochondria: (i) electron leakage from the transfer chain can react with oxygen-generating RONS, thus perpetuating and amplifying proximal; (ii) mtDNA is particularly susceptible to damage due to its proximity to the electron transfer chain, and (iii) mitochondria lack effective mechanisms for mtDNA repair and protection.

Under physiological conditions, RONS production is neutralized by endogenous antioxidant factors such as Manganese superoxide dismutase and glutathione. However, many factors can disrupt this balance, such as diet, injury, illness, and age. For example, the “free radical theory of aging” (Harman, 1956), now commonly referred to as the “oxidative damage theory” (Gladyshev, 2014), posits that aging itself is the result of an oxidative stress-favoring imbalance of a tripartite relationship between RONS generation, antioxidant defenses, and repair from oxidative damage (Beckman and Ames, 1998). Indeed, many have shown that in aging, the vulnerability of mtDNA to damage increases due to reductions of antioxidant defense mechanisms (Chakrabarti et al., 2011; Kubben et al., 2016). It is noteworthy that this assertion has been under continued scrutiny (Beckman and Ames, 1998; Payne and Chinnery, 2015), likely due to multiple potential avenues of mtDNA dysfunction, for example, age-related mutation of mtDNA (Bandy and Davison, 1990; Arnheim and Cortopassi, 1992). That said, such alterations frequently coincide with increased levels of free radicals, which can perpetuate the aforementioned imbalance and result in elevations of oxidative stress. Almost a decade ago, a therapeutic approach for PD targeting the mitochondrial dysfunction was reported. This study consisted of a double-blind, placebo-controlled study to assess the effect of the antioxidant MitoQ in PD pathology progression, and it was the first clinical trial of a mitochondria-targeted antioxidant (Snow et al., 2010; Chaturvedi and Beal, 2013).

Increased RONS production and/or decreased neutralization can also cause neuronal death through lipid peroxidation and oxidation and nitration of proteins (Keane et al., 2011; Videira and Castro-Caldas, 2018). Together, these processes can then trigger apoptotic signaling leading to mitochondrial dysfunction. Indeed, RONS-initiated mitochondrial dysfunction accelerates the damage and death of dopaminergic neurons (Keane et al., 2011; Bose and Beal, 2016). Thus, energy and mitochondrial dysfunction is the earliest modifiable defect in the aging brain, and treatment with agents that improve mitochondrial function or enhance antioxidant activity may be beneficial in neurodegenerative diseases (Beal, 2005). A recent study investigating the role of telomerase in neuronal degeneration reported a new mechanism of mitochondrial dysfunction. Kim H. et al. (2017) used CRISP9/Cas9 technology to eliminate telomere repeats in the Neuroblastoma cells SH-SY5Y. Telomere removal resulted in mitochondrial dysfunction that adversely affected mitochondrial respiration and cell viability. Telomere removal also altered the levels of various PD-associated proteins, including PTEN-induced putative kinase 1, peroxisome proliferator-activated receptor gamma coactivator 1-alpha, nuclear respiratory factor 1, parkin, and aminoacyl tRNA synthetase complex interacting multifunctional protein 2. Finally, telomere removal enhanced α-synuclein protein aggregation, suggesting that this mechanism may be one of the links between aging and PD (Kim H. et al., 2017).

### Dopaminergic Input and Organizational Features of the Dorsal and Lateral Striatum

As reviewed above, it is generally accepted that dysfunction in PD stems from the degeneration of SNc neurons (i.e., nigrostriatal pathway), which leads to motor dysfunction and the loss of VTA neurons (i.e., mesolimbic and mesocortical pathways), which leads to behavioral dysregulation, including demotivation, anhedonia, and depression within PD (Thobois et al., 2010; Blonder and Slevin, 2011; Drui et al., 2014). While both pathways have been studied extensively across an array of conditions and pathologies, the modulatory mechanisms of the nigrostriatal pathway neurons have been fairly well described while the varied mechanisms and roles of VTA efferents continue to be elucidated. Within the nigrostriatal pathway, GABAergic medium spiny neurons (MSN) of the dorsal/lateral striatum receive excitatory glutamatergic signals that can be modulated *via* dopaminergic inputs originating from the SNc. MSNs are moderately sized cells with large, multi-structured dendritic arbors that constitute a staggering 95% of all postsynaptic nigrostriatal neurons (Kemp and Powell, 1971). Local circuit interneurons of the dorsal striatum are also actively involved in regulating MSN activity (Gittis and Kreitzer, 2012) and can be subdivided into cholinergic interneurons (1–2% of all striatal cells) and aspiny GABAergic interneurons known as low-threshold, fast-spiking neurons (Lim et al., 2014). Striatal cholinergic and MSNs express several neurotransmitter receptors including the γ-aminobutyric acid (GABA), glutamate, DA, adenosine, serotonin, opioids, and substance P (NK1) receptors (Lee et al., 1997; Tzaferis and McGinty, 2001; Solbrig et al., 2002; Lim et al., 2014).

Present on MSNs is multiple functional receptors capable of binding DA. All DA receptors are G-protein coupled and are generally classified into two subgroups according to structure, function, and pharmacokinetic properties (Watts and Neve, 1997; Gerfen and Surmeier, 2011). The first often termed “D1-like receptors,” are comprised of D1 and D5 receptors subtypes and, upon DA binding, drive adenylyl cyclase and thus cyclic adenosine monophosphate (cAMP) activity. On the other hand, “D2-like receptors,” which comprise D2, D3, and D4 receptor subtypes, suppress cAMP activity, thereby producing an inhibitory effect upon DA binding. While there is some evidence that D1- and D2-like receptors can colocalize in 4–6% of dorsal and 17–30% of ventral striatal MSNs (Matamales et al., 2009; Gangarossa et al., 2013; Perreault et al., 2015), there appears to be little, if any, functional competition between receptor subtypes within the same neuron (Biezonski et al., 2015; Frederick et al., 2015). This, therefore, yields two distinct MSN subtypes that exist in approximately equal quantities (Gerfen and Surmeier, 2011). These two types of MSNs can be further organized based on their differential projection patterns. D1-like containing MSNs monosynaptically innervate SNpr and are thusly termed the “direct” pathway while D2-like containing MSNs of the “indirect” pathway project to the GPi which, in turn, innervates various interface nuclei of the basal ganglia (Gerfen and Surmeier, 2011; Leisman et al., 2012, 2014; Leisman and Melillo, 2013; Rangel-Barajas et al., 2015). MSNs of the direct pathway also synthesize dynorphin and substance P as co-transmitters. MSNs of the indirect pathway co-transmit encephalin. While some have argued, particularly following electrophysiological studies, that the complexity of DA system physiology may be due to the coexpression patterns of D1- and D2-like receptors described above on direct and indirect pathway neurons, work using bacterial artificial chromosome (BAC) transgenic mice confirms that the “murkiness” of this system is instead a consequence of the complexity of striatal circuitry (Gerfen and Surmeier, 2011).

The influence of dopaminergic cells on the brain and behavior is impressive, considering that they account for less than 1% of the total number of neurons in the brain. This is achieved due to the numerous input signals by a small number of dopaminergic neurons in the midbrain within the striatum (Nagy et al., 2006; Reig and Silberberg, 2014). Indeed, MSNs integrate, and DA modulates signals from the cortex, thalamus, hippocampus, midbrain, brain stem, and various limbic structures (Plenz and Wickens, 2016).

**Figure 2:**
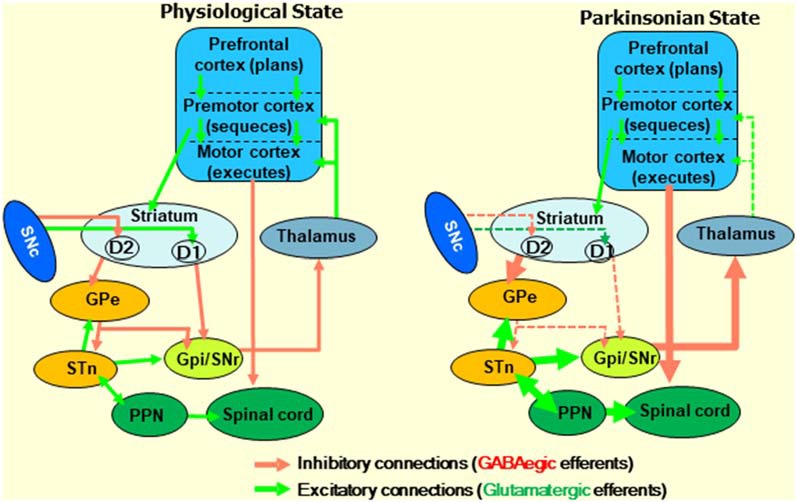
Diagram describing the frontostriatal motor loop controlling motor function under physiological and parkinsonian states. The prefrontal cortex (PFC) participates in cognitive control and planning of movements. The premotor cortex organizes sequences of body actions, and the primary motor cortex is responsible for executing them. Excitatory signals, which are initiated by cortical glutamatergic neurons, project from the PFC to the premotor cortex, and then to the motor cortex through several subcortical structures. Then, the resultant signals received by the pyramidal cells of the motor cortex go to the motor neurons of the spinal cord. Together, this is called the frontostriatal motor loop. Midbrain dopaminergic neurons play an essential role in modulating the signals that go along the frontostriatal motor loop. Changes in the direct inhibitory (initiated by D2 receptors) and indirect (D1 receptors) pathways under parkinsonian states due to the loss of dopaminergic neurons in the SNc are indicated. GABA, γ-aminobutyric acid; SNc, substantia nigra pars compacta; GPe, globus pallidus external; GPi, globus pallidus internal; STN, subthalamic nucleus; PPN, peripeduncular nucleus.

While each integrated signal within the striatum plays its critical role, inputs from the frontal cortex are particularly crucial in goal-directed movements. For example, while motoric information from the premotor cortex (PrC) converges with situational reward information from the dopaminergic system in MSNs, all three of these systems are predominantly orchestrated *via* input from the prefrontal cortex (PFC; Figure 2; Deutch, 1993; Vogelsang and D’Esposito, 2018). With a panoply of roles in the top-down regulation of emotion, cognition, and goal-directed planning, various subregions of the PFC participate in the cognitive control and planning of movements. By projecting to the PrC, the PFC organizes, and the PrC then sequences voluntary bodily actions. The PrC then projects to the primary motor cortex, which is responsible for executing the associated movements. That having been said, movements initiated by this pathway are further refined by sub-second, situational updates that loop back to the PFC, PrC, and primary motor cortex *via* several subcortical pathways. Simultaneous glutamatergic projections from the PFC and PrC to the striatum are subjected to conditional modulation *via* SNc DA, which in turn projects *via* the direct and indirect pathways that loop back to the PrC and primary motor cortices by way of interface nuclei of the basal ganglia and, finally, the thalamus. These pathways are jointly referred to as the frontostriatal circuit or motor loops. The schematic diagram in Figure 2 describes the basal ganglia circuitry involved in voluntary motor control affected in PD (Ray and Strafella, 2010).

**Figure 3:**
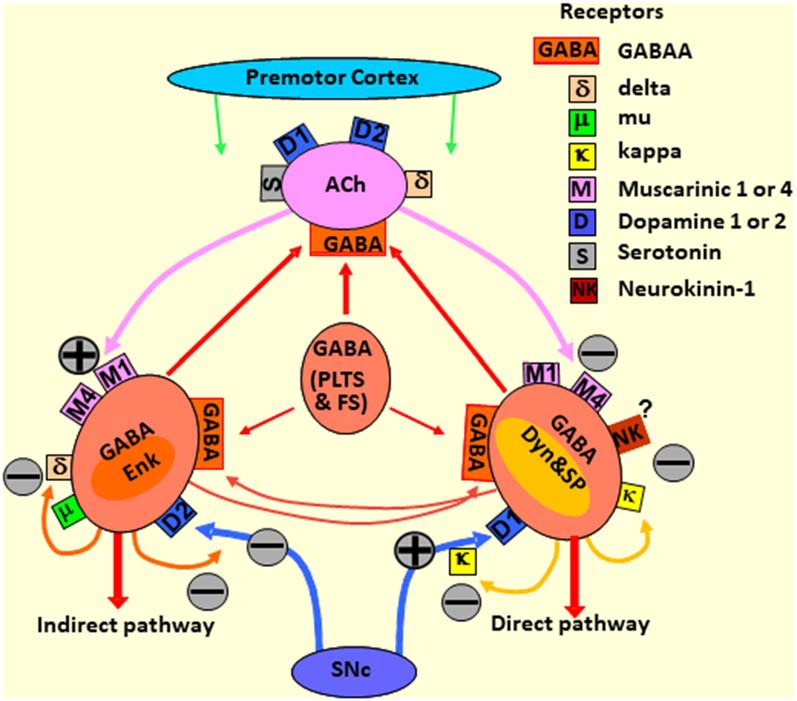
Diagram depicting the striatal neurotransmitter systems modulating the responses of the striatal neurons to the premotor cortex and SN afferent signals. The direct and indirect pathways, as well as stimulating and inhibitory neurotransmitter receptors, are outlined. MSNs, medium spiny neurons; GABA, γ-aminobutyric acid; SNc, substantia nigra pars compacta; ACh, acetylcholine; Enk, enkephalin; Dyn, dynorphin; GABA (PLTS and FS), aspiny GABAergic interneurons, low-threshold spiking (PLTS) and fast-spiking (FS) neurons.

As alluded to above, the dorsal striatum controls and modulates signals passing from the PFC and PrC areas toward the motor cortex through the striatal-motor loop. The complexity of this process is underscored by the integration and signal modulation of more than a dozen neurotransmitter systems and their receptors (Figure 3). Accordingly, dopaminergic cell death in PD is associated with massive disruptions in the flow of information coming from the midbrain system and leading to an imbalance in the action of the direct and indirect pathways (Joyce, 2001; Alexander, 2004). However, the integrated use of these various other neurotransmitter systems provides an opportunity for targeted treatments in PD.

## Progress in the Treatment of Parkinson’s Disease

Despite the fact that 200 years passed since the discovery of PD, it was not until later in the 20th century that progress in the treatment of PD was achieved, predominantly due to the limited understanding of PD pathophysiology. Given Carlsson’s discoveries of DA’s involvement in the 1950s, it became clear that PD development involved dopaminergic cell death and a decrease of DA in the striatum and other structures of the forebrain. The first steps towards treatment were made by Carlsson (2001), who proposed targeting this DA deficiency to facilitate symptom reduction.

### Treatment of Parkinson’s Symptoms With the Dopamine Precursor, L-DOPA

Based on Carlsson’s discoveries, Hornykiewicz and colleagues developed the treatment of PD with the DA precursor, L-DOPA (Lees et al., 2015). This approach compensates for decreased DA by promoting DA synthesis in midbrain DA neurons. As evidenced in several pop-culture pieces, such as the award-winning motion picture *Awakenings* starring Robin Williams and Robert De Niro and based on the novel of the same name written by Oliver Sacks, the success of this approach in patients with PD was dramatic and often quite rapid (Birkmayer and Hornykiewicz, 1961). Despite these dramatic effects, it was reported that L-DOPA’s effects were often inconsistent, even within the same patients, and often eventually induced profound and intolerable side effects such as dyskinesia, motor fluctuations, and various emotional disturbances and psychiatric problems (Allan, 2007; Voon and Fox, 2007; Fox and Lang, 2008; Salat and Tolosa, 2013). Furthermore, all the clinical benefits of the treatment are eventually reverted with a continuation of dopaminergic neuronal death, as L-DOPA administration does not halt disease progression (Castrioto et al., 2013). However, despite these limitations, the improvement seen in some patients is so pronounced that these downsides do not prevent its use. Indeed, after almost 60 years, L-DOPA remains the gold-standard medication for PD (Tan, 2001; Salat and Tolosa, 2013).

### Deep Brain Stimulation

In the late 1990s, the theory of the dual organization of the striatum and its outputs to other parts of the basal ganglia (Albin et al., 1989; DeLong, 1990; Figure 3) led to a renaissance of neurosurgical procedures for the treatment of PD. Based on this theory, a new surgical treatment to reduce the symptoms of PD called deep brain stimulation (DBS) was established (Lozano and Snyder, 2008; Lozano et al., 2010). Despite its highly invasive nature, DBS led to significant improvements in the quality of life of patients with advanced PD and consists of the direct electrostimulation of the subthalamic nucleus (STN) or the GPi that are hyperactivated due to the decrease in midbrain dopaminergic neurons.

In general, among PD patients, before surgery, women with PD use lower doses of dopaminergic medication and experienced more dyskinesias, mobility limitations, and sensory symptoms than men (Hariz et al., 2013). Nonetheless, after DBS, both sexes show a similar functional improvement (Hariz et al., 2013). Although it is effective at motor symptom alleviation, DBS can also induce adverse side effects such as an aggravation of freezing of gait and worsening of verbal fluency (Carlson et al., 2014; Foley et al., 2017; Højlund et al., 2017). Yet another downside of DBS is that PD-induced speech disruption is much less responsive to DBS than any other motor dysfunctions (Limousin and Martinez-Torres, 2008; Moro et al., 2010). The mechanism underlying the beneficial effect of DBS is not completely understood; however, the actual evidence suggests that vascular changes may be involved in its therapeutic effects (Pienaar et al., 2015). The overexpression of the vascular endothelial growth factor (VEGF) and the downregulation of neuroinflammatory factors are considered to be key molecular mechanisms involved in DBS-induced microvascular changes (Pienaar et al., 2015; Sharma et al., 2016; Lozano et al., 2018).

L-DOPA and DBS treatments are frequently applied together to potentiate their beneficial effects (Pienaar et al., 2015; Sharma et al., 2016; Lozano et al., 2018). This said, although these treatments can efficaciously reduce PD symptomology, they are ineffective at halting PD progression. Still, L-DOPA and DBS have been widely used for some decades now, thus improving the lives of patients with PD where few other options are available (Timpka et al., 2016).

### Nigral Cell Transplantation

Another promising therapeutic strategy for PD is cell replacement therapy to restore dopaminergic neurons (Kupsch et al., 1995; Stoker et al., 2017). This strategy, supported by reports in the late 1970s and early 1980s, showed that dopaminergic neurons derived from the developing embryonic midbrain were able to survive when implanted in adult brains and, moreover, that the transplanted neurons formed axons that extended through surrounding scar tissue and thus restored innervation in the host brain (Björklund et al., 1976; Stenevi et al., 1976). More recent investigations in animal models have focused on the survival and differentiation of implanted cells. This includes fetal dopaminergic neurons (Thompson and Parish, 2013) as well as embryonic, adult, neural, mesenchymal, and induced pluripotent stem cells (Freed et al., 2003; Hedlund and Perlmann, 2009; Thompson and Parish, 2013; Barker et al., 2016; Xu et al., 2016; Venkatesh and Sen, 2017; Zhang et al., 2017). These efforts were accompanied by the development of biomaterial scaffolds to provide support materials for cell adhesion and growth (Moriarty et al., 2019).

The first clinical trials were conducted in the late 1980s to investigate the transplantation of embryonic mesencephalic tissues into the striatum but resulted in only minimal clinical improvements (Drucker-Colín et al., 1988; Iriarte et al., 1988; Madrazo et al., 1988a,b). However, after refining the technique, subsequent clinical studies showed significant clinical improvements following implantation of fetal DA neurons into the brains of young PD patients (Freed et al., 2001). Unfortunately, this approach resulted in high morbidity and mortality rate in elderly patients (Madrazo et al., 1988b; Goetz et al., 1989; Brundin et al., 2010). However, postmortem studies showed a sustained survival of the transplanted cells (Barrow, 2015; Björklund and Lindvall, 2017). These studies revealed that cell replacement is a promising approach for the treatment of PD, but with many problems that remain unresolved. A significant issue has been the absence of significant and consistent therapeutic effects in patients with PD. This failure results in a 10-year moratorium for this kind of surgery, which was imposed in 2003 (Han et al., 2015). Also, other ethical and logistical problems exist, including the absence of sufficient source material for tissue transplantation for a large number of patients (Barker et al., 2016), inconvenient side effects such as graft-induced dyskinesia (Lindvall and Björklund, 2004; Lindvall, 2015, 2016), and the enhanced risk of tumor formation (Wolff et al., 2015). Nonetheless, the biology of stem cells has advanced significantly over the past decade, as has the obtention of dopaminergic neurons from neural and induced pluripotent stem cells (Björklund and Lindvall, 2017). These advances aid in resolving problems of availability as well as the rejection of transplanted cells while circumventing ethical issues associated with obtaining stem cells from embryos.

While promising, another problem with this approach resides in the complicated architecture of the midbrain dopaminergic system. As mentioned above, there are two prominent populations of dopaminergic neurons in the midbrain (i.e., SNc vs. VTA). While the loss of dopaminergic neurons in the SNc is primarily associated with motor impairment, the loss in the VTA may induce psychological and emotional disturbances (Thobois et al., 2010; Blonder and Slevin, 2011; Drui et al., 2014). In most clinical studies, stem cells were grafted directly in the striatum or the lateral ventricle. Importantly, dopaminergic neurons have complex relationships with glial and other neural cells that support their survival and activity including the formation of tripartite synapses with astrocytes in the midbrain (Hennigan et al., 2015; Xin et al., 2019). An ongoing clinical trial (NCT01898390) named The Transeuro Transplant study consists in grafting fetal tissue into the brain of patients with PD. The last update informed that 11 PD patients were subjected to cell transplantation in the UK and Sweden. The study will finish the clinical evaluation of all these patients in 2021.

These studies raise important questions in need of addressing: (i) can grafted stem cells retain their functions outside SNc and VTA; and (ii) can the new cells correctly replace the damaged ones by integrating into the existing information networks of the brain? New answers for these questions continue to appear. A more detailed description of more than 30 years of neuronal transplantation studies in PD, can be found in excellent reviews (Björklund and Lindvall, 2000; Lindvall and Björklund, 2004; Lindvall, 2015; Lindstrom, 1997; Stoker et al., 2017). Despite the challenges associated with this therapeutic approach, the considerable progress in the field predicts essential advances in the years to come.

## Preventative Approaches: Targeting the Causes of Parkinson’s Disease

Unfortunately, although some therapies for PD produce a period of recovery for about 5 years, there is a sharp decrease in the beneficial effects of treatments thereafter (Castrioto et al., 2013). Indeed, the best approach would be to understand the relevant triggers of the disease in order to target the physiopathological mechanisms causing the death of dopaminergic neurons. Epidemiological studies have shown that less than 10% of PD cases have a strict familial etiology, while most of them are sporadic and appear to be caused by other factors associated with susceptibility genes (Thomas and Beal, 2007; Videira and Castro-Caldas, 2018). Although these factors are not fully understood, there is a consensus that PD is induced by a combination of age, gender, genetic background, and environmental factors. However, neither of these has, alone, been identified as a leading cause of PD (Allam et al., 2005; Thomas and Beal, 2007; Wirdefeldt et al., 2011). While the cellular and neurochemical mechanisms underlying PD have remained incompletely understood, what data have been collected point to heavily to mitochondrial dysfunction, oxidative stress, inflammation, and excitotoxicity in the pathogenesis of both familial and sporadic cases of PD (Ouchi et al., 2009).

Despite low heritability rates, as discussed above, some rare familial forms of PD give important clues regarding the molecular mechanisms of the sporadic form of PD pathology. Currently, 28 chromosomal regions have been linked to PD; from these, six contain genes in which a single mutation causes monogenic forms of PD (3–5% of all cases; Klein and Westenberger, 2012; Videira and Castro-Caldas, 2018). These mutations affect genes responsible for autosomal dominant forms of PD including genes such as α-synuclein, Park 1/4 (SNCA), Park 8 (the Leucine-Rich Repeat Kinase 2, LRRK2), and genes exhibiting an autosomal recessive mode of inheritance such as Parkin, PINK1, DJ-1, and ATP13A2. The remaining cases of PD seem to be the result of complex gene-environmental interactions influencing the development of the disease (Klein and Westenberger, 2012).

The α-synuclein gene encodes a small protein present in nerve terminals. The physiological function and role of α-synuclein in the etiology of PD are still unclear. However, evidence suggests that this protein plays a significant role in the pathogenesis of the disease (Schapira and Jenner, 2011). Because of its structure, α-synuclein can interact with anionic lipids, which results in conformational changes favoring aggregation into toxic complexes. Also, these aggregate-prone forms of α-synuclein can interfere with lysosomal and mitochondrial functions, autophagy, vesicular homeostasis, and microtubule transport (Keane et al., 2011; Rocha et al., 2018). For example, the accumulation of mutant forms of α-synuclein in the inner mitochondrial membrane impairs the complex I, increasing RONS production and promoting neuronal death (Devi et al., 2008).

Interestingly, all familial forms of PD are associated with mutations in genes that directly or indirectly cause mitochondrial dysfunction. Often these mutations have multiple pathological effects on mitochondria. For example, mutations in the genes SNCA, Parkin, DJ-1 inhibit the activity of the complexes I, II, and III and affect mechanisms regulating the morphology and dynamics of mitochondria (Beal, 2005; Moon and Paek, 2015; Ryan et al., 2015; Bose and Beal, 2016). These disturbances in the mitochondria or direct inhibition of its complexes in both sporadic and familial PD affects mitochondrial integrity and induce negative bioenergetic effects, such as a dysregulation of glucose metabolism, impaired pentose phosphate pathway, and a decrease in ATP production (Dunn et al., 2014). The complex I of damaged mitochondria vigorously produces RONS, and when the production exceeds the cell antioxidant capacity, the oxidant species damage mitochondrial proteins, lipids, and mtDNA. Damage to mtDNA leads to mutations that inhibit the respiratory chain and reduce the mitochondrial membrane potential (Ryan et al., 2016). Ultimately, these self-reinforcing processes lead to proliferative cell death.

This evidence suggests that preventing mitochondrial dysfunction can be a key therapeutic goal to achieve as stand-alone or adjunctive therapy against PD.

### Genetic Approaches

#### CRISPR Technology

The explosive development of new genetic editing technologies, although still under investigation for clinical use, open the possibility to correct mutated genes and regulatory DNA in the monogenic forms of PD (Nadim et al., 2017; Singh and Sen, 2017; Deverman et al., 2018; Kabra et al., 2018; Lu et al., 2018). With these ideas in mind, several methods of gene delivery, including viral vectors and CRISPR, have been developed (Lino et al., 2018). Outstandingly, new reports for clinical trials have shown success in CRISPR technology use against diseases induced by single mutations such as β-thalassemia and sickle cell anemia. However, because Cas9 induces a double-strand DNA break, potential detrimental side effects of CRISPR-Cas9 technology are possible, such as non-specific CRISPR-induced mutation due to deletions in non-intended regions of the genome (Ihry et al., 2018). A description of PD linkage studies using CRISPR technology as well as the process of genome editing in PD patients’ inducible progenitor stem cells (iPSCs) has been reviewed and reported (Safari et al., 2019).

Active research efforts are currently underway to overcome these limitations. Recently one of the latest advances in CRISP technology was reported by Rees et al. (2019) from Harvard University (Anzalone et al., 2019). In this new approach, Cas9 hybridizes to the target DNA site using a guide engineered RNA containing a complementary spacer. To transfer the latest information from these guide RNAs, the genomic DNA is nicked at only one location (Anzalone et al., 2019). This method reduces the risk of undesired DNA mutations, and it may very well revolutionize the therapy of PD and other pathologies linked to single-gene mutations.

### Viral Vectors

Another promising approach lies in gene therapy using non-replicating viral vectors such as gene delivery forms of adeno-associated virus (AAVs), retro and lentiviruses (Lundberg et al., 2008), and glycoprotein-deleted rabies virus (Chen et al., 2019; Wang and Huang, 2019; Wang F. et al., 2019; Wang X. et al., 2019; Wang Y. et al., 2019). Gene delivery using AAVs has the advantage in that these viruses do not integrate into host chromosomes yet persist as episomic chromosomes that do not provoke insertional mutations and permit stable gene expression in neuronal and glial cells (Penaud-Budloo et al., 2008). Furthermore, AAVs do not induce immunoreactions in humans and, as a result, are regarded as one of the best viral gene delivery systems for use in preclinical biomedical research and clinical trials (Naso et al., 2017). The main limitation of AAVs is that they can only deliver up to 5.2 kb of genetic material (Wu et al., 2010). Lentiviruses, however, can deliver genetic sequences of up to 9 kb to dividing and non-dividing cells. After transduction, the lentiviral RNA is reverse transcribed to DNA and randomly integrated into the host chromosomes (Rodríguez et al., 2019). This disadvantage limits its clinical application though lentiviruses are frequently used in preclinical research (Maes et al., 2019).

The recent approval of human AAV vector use in Europe and the USA has led to an array of gene therapy attempts in various clinical trials (Piguet et al., 2017; Axelsen and Woldbye, 2018; Hitti et al., 2019). The genetic approaches taken for PD treatment are largely neuro-regenerative in nature, and they are directed to halt neuronal cell death. For example, some strategies include inducing the overexpression of neurotrophic factors in the substantia nigra or the increasing repair genes to disrupt the formation and accumulation of aggregated and neurotoxic forms of neuronal proteins such as a-synuclein. More than a decade ago, a pioneering phase 1 study assessing the safety of human aromatic L-amino acid decarboxylase (hAADC) gene therapy for PD tested the effect of bilateral AAV2-induced AADC expression in the putamen of subjects with advanced PD (Eberling et al., 2008). Although the authors reported no adverse effects of AAV-mediated AADC overexpression in humans, they found no significant clinical recovery as tested using the Unified PD Rating Scale (UPDRS; Eberling et al., 2008). Follow-up clinical studies reported positive effects, such as reduction of symptoms and improvement of UPDRS scores, as well as lowered L-DOPA dosage required for treatment. Other recent studies include the Phase 1 trial and current Phase II trial for Voyager’s AAV2-hAADC transplantation, a year-long clinical trial in PD patients that also investigates changes in overnight time free of dyskinesia (McFarthing et al., 2019). Furthermore, gene therapy has been used with the intent to prevent mitochondrial dysfunction in the brain of patients with PD, by increasing the expression of synaptic proteins, neurotrophic factors (NTFs), antioxidants, and anti-inflammatory proteins.

### Neurotrophic Factors

The overexpression of neurotrophic factors (NTF) is a powerful strategy to prevent the neurodegeneration of dopaminergic neurons in PD brains. The delivery of these factors, including the neurotrophic factor (NF), glial cell line-derived neurotrophic factor (GDNF), neurturin (NRTN), cerebral dopamine neurotrophic factor (CDNF) and growth/differentiation factor 5 (GDF5) is a challenging task. An alternative approach is the use of recombinant viral vectors to enable long-term expression of these factors in brain cells without the risk of hemorrhages induced by the catheter placement into the brain. From them, genetic therapy directed to increase the expression of GDNF and NRTN alone or combined with other NFs have shown promising results.

The study of GDNF both *in vitro* and *in vivo* using rodent (Rosenblad et al., 1998; Sullivan et al., 1998; Georgievska et al., 2002) and monkey models of PD (Gash et al., 1996; Miyoshi et al., 1997; Palfi et al., 2002; Eberling et al., 2009; Su et al., 2009; Redmond et al., 2013), revealed potential therapeutic effects that encouraged its clinical investigation. These benefits included behavioral improvements and protective effect on the dopaminergic nigrostriatal neurons (Eslamboli et al., 2005; Sun et al., 2005; Sajadi et al., 2006; Eberling et al., 2009) However, GDNF therapy was not initially successful due predominantly to the low efficiency of delivery methods (Lang et al., 2006). Accordingly, subsequent efforts were refocused on improving delivery and expression methods, including; infusion, cannula design, and insertion zones to optimize the delivery of AAV vectors expressing GDNF to the brain (Lang et al., 2006; Richardson et al., 2011).

Other study tested AAV2-delivered NRTN, under the name CERE-120 (Ceregene Incorporated; Marks et al., 2010). While animal models of PD and an open-label, phase I clinical trial suggested tolerability and a favorable safety profile as well as reductions of UPDRS scores and dyskinesias, a follow-up phase II trial with bilateral intraputaminal injections resulted in nearly a third of CERE-120-treated patients reporting serious adverse events, including surgery-related complaints in many and tumor formation in three of the CERE-120-treated patients, and in two control subjects. Furthermore, treatment with CERE-120 for a year did not improve the UPDRS scores of the PD patients when compared to the control sham-operated group. However, CERE-120-treated patients did show significant improvements in off-medication UPDRS scores when assessed after 18 months, suggesting a delayed neurotrophic effect (Marks et al., 2010).

Another open-label trial assessed the safety and efficacy of targeting both the SNpc and striatum. Patients received nigral and putaminal doses of CERE-120 and were monitored for 2 years. This trial reported no treatment-induced adverse medical events during the entire study (Bartus et al., 2013). In the follow-up, placebo-controlled, double-blind phase 2b study, the data did not show significant improvements in motor-off scores. However, significant motor-off improvements were observed in patients that had been diagnosed less than 5 years before treatments when compared with those who were diagnosed more than 10 years before gene therapy interventions began. This indicates that gene therapy can be useful as a preventative approach in PD (Bartus et al., 2014).

A more recent study reported the results of a 6-month double-blind, randomized trial assessing the clinical effects of bilateral brain delivery of the glutamic acid decarboxylase (GAD) gene into the subthalamic nuclei (STN) of advanced PD patients (LeWitt et al., 2011). The results showed significant improvements in the UPDRS scores in the AAV2-GAD group compared with the sham group at 6 and 12 months. Also, the levodopa-induced dyskinesia significantly diminished in duration in the AAV2-GAD group, but not in the control group that remained constant. On the other hand, functional network connectivity analysis showed an increase in the metabolism of the network after a year from baseline, as investigated by PET imaging. Specifically, enhanced metabolic activity was observed in the premotor cortex, motor cortex, and supramarginal gyrus. Reduced metabolic activity is observed in the putamen, caudate, globus pallidus, inferior frontal gyrus, medial dorsal thalamus, and ventral anterior thalamus. The beneficial effects persisted when investigated 12 months after interventions (Niethammer et al., 2017).

Recently, another study reported positive effects using a lentiviral vector named AXO-Lenti-PD, which encodes three enzymes essential for DA synthesis (aromatic L-amino acid decarboxylase; cyclohydrolase 1; and tyrosine hydroxylase). Three months into the ongoing phase II trial of AXO-Lenti-PD, initial reports show a 25-point reduction in UPDRS scores. The study (NCT03720418) consists of two parts. In the first part, two patients with advanced PD who received a one-time administration of the lowest dose of AXO-Lenti-PD, which was reportedly well-tolerated and safe. A new cohort with up to six patients will receive three times the initial dose. The Initial data from this cohort is expected by the end of 2019.

Additional gene therapy approaches can be directed to knockdown the expression of PD-related genes using small interfering RNA (siRNA) or microRNA (miRNA). Various groups have focused on suppressing α-synuclein expression in animal models using RNAi (Zharikov et al., 2015; Kim Y.-C. et al., 2017. These groups have shown that reducing α-synuclein expression leads to reductions in dopaminergic neuron loss alongside fewer motor deficits. Thought, it is crucial to consider that blocking the expression of α-synuclein may interfere with the normal function of this protein (Surguchev and Surguchov, 2017; Burré et al., 2018; Sorrentino et al., 2019; Taguchi et al., 2019).

## Natural Products

### Green Tea and Coffee to Reduce the Risk of Developing PD

Green tea is prepared from the leaves of the *Camellia Sinensis* plant and contains phenolic compounds such as (-)-Epigallocatechin-3-gallate a potent antioxidant and neuroprotective compound. Preclinical clinical and self-report studies suggest that green tea may prevent PD (Kandinov et al., 2009; Bitu Pinto et al., 2015). However, the therapeutic mechanism of green tea’s potential protective actions in PD is unclear. It is feasible that green tea’s phenolic compounds are modulating critical neuroprotective signaling pathways in the brain (Jurado-Coronel et al., 2016b). On the other hand, green tea could exert its effects *via* caffeine-induced inactivation of the adenosine receptor.

Almost two decades ago, a clinical study investigating the relationship between coffee consumption and the risk of developing PD found that coffee intake negatively correlated with PD in a dose-dependent manner (Ross et al., 2000). PD incidence declined from 10.4 per 10,000 person-years in male subjects who consume no coffee to 1.9 per 10,000 people/year in men who drink at least 28 oz)approximately three cups) of coffee a day. The authors concluded that higher coffee and caffeine intake are associated with a significantly lower risk of developing PD (Ross et al., 2000). Numerous studies have since confirmed these findings and point to adenosine A2A receptor antagonists as a putative treatment for PD (Schwarzschild et al., 2002; Kalda et al., 2006; Postuma et al., 2012, 2017). However, a recent study concluded that in PD patients, consumption of 200 mg coffee a day for 6 months did not improve the motor symptoms. Still, other clinical studies using the A2A receptor antagonist, istradefylline, are encouraging given that istradefylline significantly improved motor manifestations of PD and reduced nighttime urinary frequency (Jankovic, 2008; Matsuura and Tomimoto, 2015; Kitta et al., 2018).

## Correcting Cholinergic Deficits in Parkinson’s Disease: Cotinine a Potential Therapeutic Agent

The cholinergic system plays a broad role in controlling neurotransmitter release, reducing neuroinflammation, and promoting neuronal survival and synaptic plasticity in the brain. The binding of acetylcholine (ACh) to nicotinic ACh receptors (nAChRs) occurs throughout the brain, including within striatum and other constituents of the mesolimbic, mesocortical, nigrostriatal, and frontostriatal loops. nAChRs are pentameric ligand-gated ion channels composed of α-subunits (α2-α7) or containing α and β-subunits (β2–β4; Quik et al., 2007). Presynaptic nAChRs mediate neurotransmitter release and postsynaptic receptors increase neuronal firing rates and thus facilitate long-term potentiation.

As discussed above, the striatum contains large aspiny cholinergic interneurons (ChIs) that interact with DA inputs. While ChIs is the primary source for ACh in the striatum, cholinergic projections also arrive from the pedunculopontine nucleus (PPN) and the laterodorsal tegmental nuclei (Tanimura et al., 2018). DA depletion in the striatum causes increased the excitability of ChIs as a consequence of the loss of the inhibitory dopaminergic modulation *via* presynaptic D2-like receptors on ChIs. Deficits in ChI function is involved in various basal ganglia-related movement disorders such as dystonia, PD, and Tourette’s syndrome (Pisani et al., 2007; Deffains and Bergman, 2015; Tanimura et al., 2018). Striatal ChIs appear to support synaptic plasticity and cognitive functions, mediated by the dorsal striatum such as attention, and motivation (Bohnen and Albin, 2011; Deffains and Bergman, 2015; Aarsland, 2016; Aarsland et al., 2017; Schapira et al., 2017a). ChIs and DA work together to regulate motor function and represent good targets to alleviate PD symptoms (Ztaou and Amalric, 2019). In the striatum, ChIs express the muscarinic acetylcholine receptors (mAChRs; M1/M5) as well as various subtypes of nAChRs, composed mainly of α4, α6, α7, β2, and β3 subunits, with the primary expression of the α4β2 and α6β2 receptors (Quik and Wonnacott, 2011). Striatal nAChRs are expressed in dopaminergic and glutamatergic neurons, as well as ChIs and GABAergic interneurons (English et al., 2011; Nelson et al., 2014). On the other hand, nAChRs are absent from MSNs (Quik et al., 2007).

Several studies have investigated changes in the expression of muscarinic and nAChRs in PD (James and Nordberg, 1995; Lindstrom, 1997; Quik and Jeyarasasingam, 2000; Forgacs and Bodis-Wollner, 2004; Picciotto and Zoli, 2008; Shimohama, 2009; Kawamata et al., 2012; Jurado-Coronel et al., 2016a; Zhao et al., 2016). Various studies showed that α4β2 and α7nAChRs were reduced in the cortical and subcortical regions of the brain, including the frontal and temporal cortices, hippocampus, caudate nucleus, and the pons of patients with PD when compared to healthy controls (Lange et al., 1993; Perry et al., 1995; Banerjee et al., 2000). It has also been reported an inverse correlation between the level of dementia and nAChRs expression in the hippocampus and temporal cortex of the PD patients with a loss or down-regulation of these receptors preceding the loss of dopaminergic neurons (Meyer et al., 2009). A significant decrease in nicotine binding (65–75%) has also been reported in the SNc of the midbrain, as well as a significant pathological change of cholinergic neurons of the pedunculopontine region (Perry et al., 1995). Other studies showed severe losses in α6β2 receptor expression and a minor decline in the α4β2 subtypes in PD brains. The decrease in α6β2, but not α7 receptors, paralleled a reduction in markers of nigrostriatal degeneration (Bohr et al., 2005). Within the putamen, there was no change in the expression of the α2-α7, β2, and β3 nicotinic subunits and the authors suggested that the observed binding deficits may be the result of a change in the assembly of the receptors’ subunits likely induced by α-synuclein instead of a change in the expression of the nicotinic subunits in the striatum (Martin-Ruiz et al., 2002). Brain imaging studies using positron emission tomography, and the α4β2 receptor-specific radioligand 2-18F-FA-85380 or (^123^I)5IA and single-photon emission computed tomography revealed a decrease in the number of nAChRs in the amygdala of patients with PD (Quirion, 1993; Pimlott et al., 2004; Fujita et al., 2006; Schmaljohann et al., 2006; Oishi et al., 2007; Meyer et al., 2009, 2014). This is similarly true in frontal and parietal cortices, the striatum, and substantia nigra in the PD brain (Kas et al., 2009). Nevertheless, a compensatory increase in the expression of the nAChRs has been identified during the early stages of the pathology (Isaias et al., 2014).

Epidemiological studies have shown lower rates of PD development in people consuming tobacco products, which suggests that the nicotinic receptors may play an essential role in preventing PD and that one or more tobacco-derived compounds may be neuroprotective (Fratiglioni and Wang, 2000; Parain et al., 2003; Hong et al., 2009). Various studies using cellular models have shown a neuroprotective effect of nicotine that diminished dopaminergic neuronal damage (Riveles et al., 2008; Toulorge et al., 2011; Getachew et al., 2019). Other reports have shown that both nicotine and its main derivative, cotinine, have a neuroprotective effect against 6-hydroxydopamine (6-OHDA)-induced toxicity in cultured differentiated SH-SY5Y neuroblastoma cells expressing nAChRs (Pogocki et al., 2007; Riveles et al., 2008).

Within *in vivo* animal models of PD, nicotine also appears to produce beneficial effects (Linert et al., 1999; Salminen et al., 1999; Quik and Kulak, 2002; Quik et al., 2006; Huang et al., 2009). Studies using non-human primates show that nicotine reduced dyskinesia in PD (Bordia et al., 2008), but not all studies have found a positive effect of transdermal nicotine on the cognitive or motor symptoms of PD (Lemay et al., 2004). It is not clear whether nicotine effects are the result of the activation or desensitization of the nAChRs. Studies using antagonists, it has been discovered that both α7 and α4/β2 nAChRs contribute to the neuroprotective properties of nicotine, although the effects of the cholinergic modulators will vary according to the type of brain cells and the different combinations of nicotinic subunits that may predominate in specific brain regions. In addition to nicotine, the neuroprotective effects of other nAChR modulators have been investigated and yield promising results (Pogocki et al., 2007; Tiwari et al., 2015; Jurado-Coronel et al., 2019). Derivatives clinically tested include Isoproniclina (TC1734), (S)-N-metil-5-(5-pirimidinil)-4-penten-2-amina (TC1827), and RJR-2403 (transmetanicotina), which were shown to stimulate DA release and improve working memory in PD patients (Pogocki et al., 2007). Additionally, SIB-1508Y, an α4β2 agonist, has been investigated in various clinical trials for PD (Pogocki et al., 2007). The results obtained collectively support the view that nAChRs modulators are neuroprotective against the neurotoxic insults in the PD brain.

Research from our and other labs has shown that nicotine’s predominant metabolite, cotinine has unique pharmacokinetic and pharmacodynamic properties, acts as a weak agonist at nAChRs, and a positive allosteric modulator of α7nAChRs, and is safe and non-addictive (Grizzell and Echeverria, 2015). Cotinine protects astrocyte from the toxic effects of glucocorticoids and increases synaptogenesis in the PFC and hippocampus during stress in mice (Grizzell et al., 2014a,b; Alvarez-Ricartes et al., 2018). Cotinine also diminished the activation of macrophagic immune cells (Rehani et al., 2008) and showed neuroprotective effects in mice models of AD, reducing plaque deposition, tau phosphorylation, and cognitive impairments (Buccafusco and Terry, 2003; Buccafusco et al., 2007; Szymańska et al., 2007; Echeverria et al., 2011; Echeverria and Zeitlin, 2012; Gao et al., 2012, 2014; Moran, 2012; Patel et al., 2014; Li et al., 2015; Terry et al., 2015; Echeverria et al., 2017; Grizzell et al., 2017). Also, cotinine seems to control the number and stoichiometry of the nAChRs affecting their properties (Lester et al., 2009).

Despite their chemical similarities, cotinine and nicotine differ in their mechanisms of action, behavioral effects, and show distinct properties and toxicity profiles. Cotinine is a hundred times less toxic than nicotine and binds poorly α7nAChR in the orthostatic site (Grizzell and Echeverria, 2015). Based in its neuroprotective effects, and its ability to increase DA levels and positively modulate the α7nAChRs in the brain, we have postulated that cotinine might also delay the development of PD (Soto-Otero et al., 2002; O’Leary et al., 2008; Riveles et al., 2008; Barreto et al., 2014).

## Precision Medicine in a Preventive Approach

The failure of current therapies for PD may be due to the heterogeneity of syndromes collectively referred to as PD. While all converge in the massive loss of DA neurons in the midbrain alongside the appearance of Lewy bodies, different etiologies of PD have been found. Some of these etiologies specifically affect the DA neurons, while others may overlap with comorbid conditions such as AD and other synucleinopathies. Based on this idea, the “multiple hit” hypothesis was proposed, in which the basis for selective neuronal death is a combination of toxic stress, induced by DA oxidation or mitochondrial dysfunction, co-occurring with inhibition of neuroprotective responses, such as follows after the loss of parkin function (Sulzer, 2007).

This said, the bright side of PDs multifactorial etiology provides an opportunity for more personalized treatment regimens. Precision medicine is driven to improve specific molecular alterations and treat particular subtypes of PD (Okun, 2017). Personalized medicine is not a novel treatment approach outside of PD, and it is currently used in an array of conditions, such as oncology and cystic fibrosis (Schilsky, 2014). The slow development of PD gives a unique opportunity to study the patient’s genome and environmental factors to target the causes of the disease in each specific group of patients (Barouki et al., 2018).

The availability of biomarkers to assess the appearance and progression of PD is fundamental to perform an early therapeutic intervention as well as to monitor the clinical response. So far there are several leading biomarker candidates including α-SYN (Atik et al., 2016), image biomarkers (Saeed et al., 2017), LRRK2 (Taymans et al., 2017), and microRNAs (Quinlan et al., 2017; Khodadadian et al., 2018).

The discovery of additional molecular biomarkers in groups of patients with different etiologies may permit the classification of PD subtypes according to clinical symptoms and differential molecular profiles. Accordingly, better profiling of individual patients with PD will allow the development of more effective therapies for specific PD subtypes, thus increasing the effectiveness and saving valuable time and resources during treatment.

## Conclusions

The development of effective preventive or curative therapies for PD has been extremely challenging. The causes may involve additive or impeding factors, including a limited understanding of the mechanisms of neurodegeneration in PD, the heterogeneity of the pathology, and lack of adequate animal models. Also, the clinical effectiveness of preventative therapies has been challenging to assess due to the limitations in trial designs and because of the absence of reliable biomarkers to diagnose the pathology at early stages before irreversible neuronal damage occurs. Despite the current restrictions, success in preventing or halting the development of PD should be possible due to the constant appearance of new diagnostic methods and the current significant advances in gene therapy and other therapeutic approaches in the field of neurology and neuroscience.

## Author Contributions

AI and VE drafted the manuscript. GB and JG participated in revisions for intellectual content.

## Conflict of Interest

The authors declare that the research was conducted in the absence of any commercial or financial relationships that could be construed as a potential conflict of interest.

## Abbreviations

5-HT: serotonin
AADC: the aromatic L-amino acid decarboxylase
AD: Alzheimer’s disease
cAMP: cyclic adenosine monophosphate
DBS: deep brain stimulation
ESC: embryonic and adult stem cells
GPi: globus pallidus internal
iPSC: induced pluripotent stem cells
LRRK2: Leucine-Rich Repeat Kinase 2
MSCs: mesenchymal stem cells
MSN: medium spiny neurons
NK1: substance P
NSC: neural stem cells
NOS: nitric oxide synthase
PD: Parkinson’s disease
RONS: reactive oxygen/nitrogen species
rRNA: ribosomal RNAs
SNc: substantia nigra *pars compacta*
SNpr: substantia nigra *pars reticulate*
STN: subthalamic nucleus
tRNA: transfer RNAs
UPDRS: the unified PD rating scale
VEGF: vascular endothelial growth factor
VTA: ventral tegmental area.
